# Magnetic resonance imaging features of gelatinous pseudocysts in cryptococcal meningoencephalitis

**DOI:** 10.1007/s13760-018-1033-6

**Published:** 2018-10-29

**Authors:** Ping Zhang, Lifei Lian, Furong Wang

**Affiliations:** Department of Neurology, Tongji Hospital, Tongji Medical College, Huazhong University of Science and Technology, No.1095 Jiefang Road, Wuhan, 430030 China

**Keywords:** Gelatinous pseudocysts, Cryptococcal meningoencephalitis, Magnetic resonance imaging

## Introduction

Cryptococcosis is the most common mycotic infection of the central nervous system (CNS). It more frequently occurs as an opportunistic infection in patients with either human immunodeficiency virus (HIV) infection or other immunocompromised conditions. CNS cryptococcosis primarily manifests as meningitis [1]. The pseudocysts and the granulomas of the choroid plexuses were reported to be specific findings on magnetic resonance imaging (MRI) [2]. We describe here a HIV seronegative case of cryptococcal meningoencephalitis presenting multiple gelatinous pseudocysts.

## Case report

**Fig. 1:**
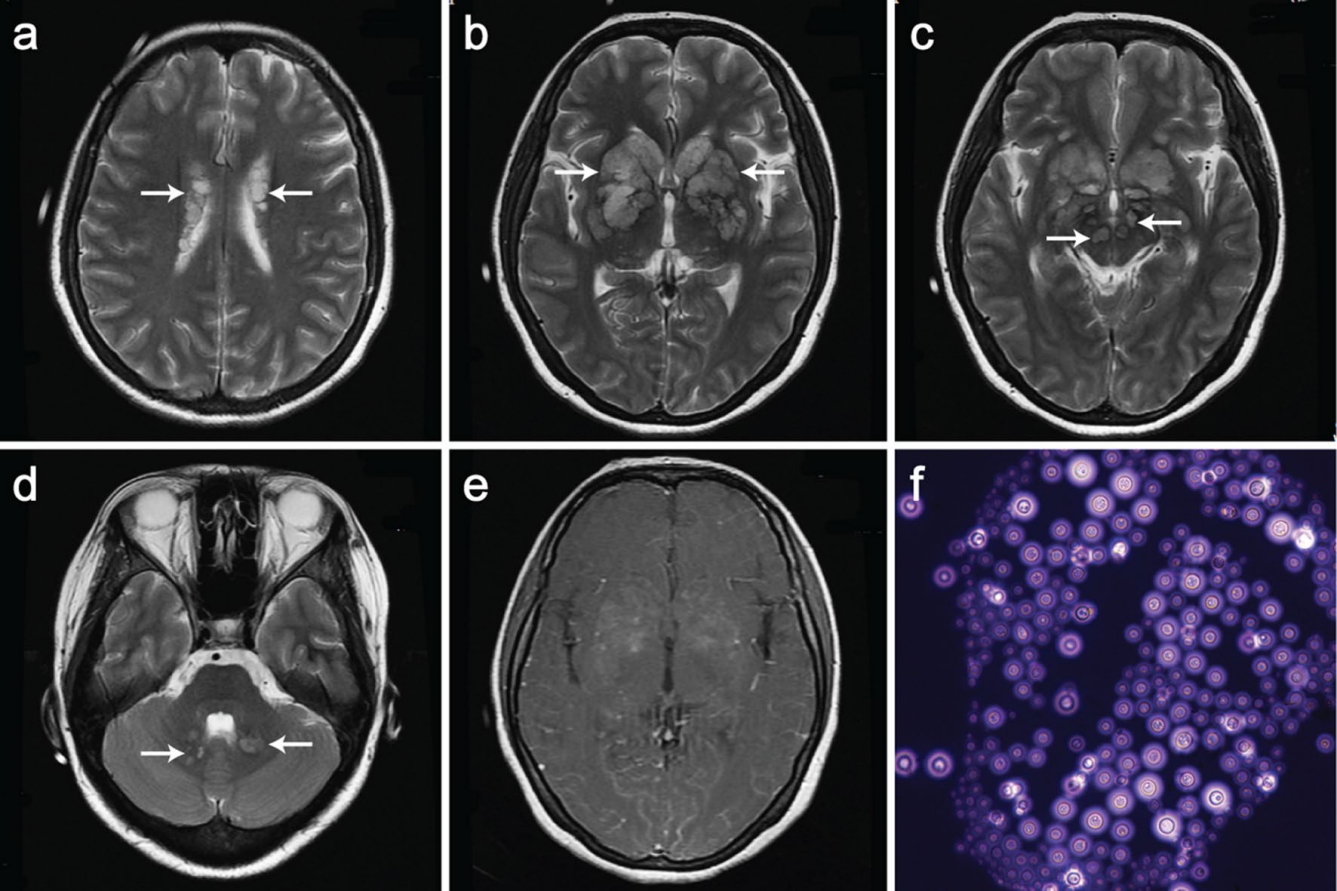
Imaging shows gelatinous pseudocysts in the brain of cryptococcal meningoencephalitis patient. T2-weighted magnetic resonance images show clusters of soap bubble-like gelatinous pseudocysts (arrows) at the periventricular white matter (**a**), basal ganglia (**b, c**), midbrain (**c**) and dentate nucleus (**d**). T1-post gadolinium shows mild enhancement at bilateral basal ganglia (**e**). India ink staining of CSF shows typical round encapsulated Cryptococcus neoformans (**f**)

A 35-year-old woman with a history of autoimmune hemolytic anemia and longtime prednisone taken presented with headache, fever and altered mental status. Neurological examination revealed meningeal irritation and bilateral pathological reflex of Babinski sign. Brain MRI showed bilateral and multiple hypointense T1 (not shown) and hyperintense T2 soap bubble-like gelatinous pseudocysts at the periventricular white matter, basal ganglia, midbrain and dentate nucleus, with mild post-gadolinium enhancement at bilateral basal ganglia (Fig. 1 a–e). Lumbar puncture was performed with cerebral spinal fluid (CSF) pressure of 350 mmH_2_O. CSF was clear with a protein level of 359 mg/L (normal range: 150–450 mg/L), a glucose level of 0.54 mmol/L (normal range 2.22–3.89 mmol/L), but without leukocytes. India ink staining of CSF found Cryptococcus neoformans (Fig. 1f) and fungal spores. Fungal culture and cryptococcal capsular polysaccharide antigen test of CSF confirmed CNS cryptococcal infection. Subsequently, blood culture also revealed cryptococcal infection. Serum HIV test of this patient was negative. However, lymphocyte count in the peripheral blood was as low as 0.03 × 10^9^/L (normal range: 1.10–3.20 × 10^9^/L). Peripheral blood lymphocyte subsets analysis revealed a significant reduction of all kinds of immune cells, especially the extremely reduced CD4 + T lymphocytes and NK cells (Table 1). Ultrasonography showed splenomegaly. Based on these findings, cryptococcal meningoencephalitis was diagnosed in this immunocompromised patient. Though received standard antifungal treatment, the patient died of cerebral hernia 3 weeks later.

**Table 1:** Peripheral blood lymphocyte subsets of the cryptococcal meningoencephalitis patient

Item	Value	Reference range	Unit
Total T lymphocytes (CD3 + CD19-) (%)	42.84	50–84	%
Total T lymphocytes (CD3 + CD19-) (#)	34	955–2860	/µl
Total B lymphocytes (CD3-CD19+) (%)	51.56	5–18	%
Total B lymphocytes (CD3-CD19+) (#)	41	90–560	/µl
Helper/induced T lymphocytes (CD3 + CD4+) (%)	9.11	27–51	%
Helper/induced T lymphocytes (CD3 + CD4+) (#)	7	550–1440	/µl
Suppressor/cytotoxic T lymphocytes (CD3 + CD8+) (%)	29.30	15–44	%
Suppressor/cytotoxic T lymphocytes (CD3 + CD8+) (#)	23	320–1250	/µl
NK cells (CD3-/CD16 + CD56+) (%)	4.43	7–40	%
NK cells (CD3-/CD16 + CD56+) (#)	3	150–1100	/µl
T lymphocytes + B lymphocytes + NK cells (%)	98.83	95.00–105.00	%
T lymphocytes + B lymphocytes + NK cells (#)	78		/µl
Th/Ts	0.31	0.71–2.78	

## Discussion

Cryptococcosis in most cases affects patients with acquired immune deficiency syndrome (AIDS). Less frequently, it can be found in other immunocompromised patients. In this case, the HIV seronegative patient had a history of autoimmune hemolytic anemia after she gave birth to her second baby. She took prednisone orally for 7 months and then stopped for 12 months before this onset. Anemia no longer existed. However, the extremely low peripheral blood lymphocyte count and the absence of leukocyte reactivity in CSF were clues of immunosuppression. Peripheral blood lymphocyte subsets analysis revealed a significant reduction of T lymphocytes and NK cells, which helped confirming immunodeficiency.

Meningitis pronounced at the base of the brain is the primary pathological lesion of CNS cryptococcosis. Occasionally the meningeal infection can extend along perivascular spaces and give rise to small cysts in the Virchow–Robin spaces and adjacent brain, which term gelatinous pseudocysts [3]. Gelatinous pseudocysts exhibit soap bubble appearance by MRI, with a low to intermediate T1WI signal, a high T2WI signal and a low T2-FLAIR signal [4, 5]. In immunocompromised patients, the enhancements of the cystic lesions and meninges are usually mild or absent. These lesions can help the diagnosis of cryptococcal meningoencephalitis.
